# Advanced Age and Multiple Comorbidities as Important Factors in Predicting Poor Prognosis in Herpes Zoster Ophthalmicus

**DOI:** 10.7759/cureus.18412

**Published:** 2021-09-30

**Authors:** Asuman Orhan Varoğlu, Aysenur Avarisli

**Affiliations:** 1 Department of Neurology, Istanbul Medeniyet University, Istanbul, TUR

**Keywords:** cranial nerves, varicella-zoster virus, prognosis, ophthalmoplegia, herpes zoster

## Abstract

The varicella-zoster virus (VZV) infection results in varicella (chickenpox) and is generally seen in immunocompromised persons. VZV virus remains latent in the ophthalmic branch in the trigeminal ganglion. When reactivated, herpes zoster ophthalmicus (HZO) develops and sometimes leads to chronic ocular complications, among which cranial nerve palsies are rarely seen. Though the third cranial nerve is most frequently involved, the fourth and sixth nerves may also be involved in some cases. Treatment includes systemic antiviral therapy and steroid administration. The prognosis is generally good when treatment is executed. Improvement can also be observed without treatment. In this article, we would like to highlight two such cases in which these two cranial nerves got involved following an episode of HZO. One is a 67-year-old female patient having diabetes mellitus (DM), hypertension (HT), and coronary heart disease with fourth and sixth cranial nerve complete palsy. The other is a 76-year-old male patient with HT, DM, and heart failure with only sixth cranial nerve complete palsy. Despite adequate treatment, both patients had a poor prognosis. Advanced age and the presence of multiple comorbidities are important factors in predicting poor prognosis in HZO cases.

## Introduction

Herpes zoster ophthalmicus (HZO) is a condition that is characterized by inflammation of the sensory fibers of the ophthalmic branch of the trigeminal nerve. The most common complications of varicella zoster are gastroenterological infections, cranial nerve palsies, myelitis, meningitis, and stroke [1]. In HZO, the incidence of extraocular muscle paralysis ranges from 7% to 31%; however, most cases can be overlooked, as visual acuity is reduced in the affected eye and diplopia is seen in extreme gaze [2]. While the oculomotor nerve is more affected, the abducens and trochlear nerves are less affected. In HZO, extraocular muscle paralysis sometimes develops at the same time as the rash develops, and sometimes more than four weeks after the rash develops; however, it usually occurs two to four weeks after the development of the rash [3]. Extraocular muscle paralysis is usually seen in elderly patients with a benign prognosis [1]. Here, unlike the literature, we present two cases of persistent extraocular muscle complete paralysis in elderly patients despite adequate treatment.

## Case presentation

Case 1

**Figure 1:**
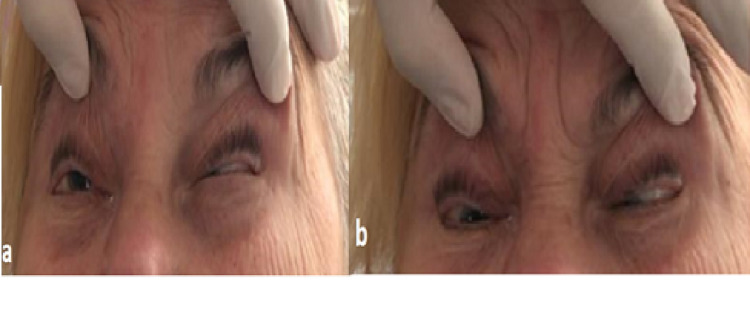
When looking to the right, the eye cannot look down and out. Fourth and sixth cranial nerve paralysis were observed.

A 67-year-old female patient visited our center with diplopia and pain in the right half of her face for five days, and blisters on her scalp and forehead for a day. She had a medical history of diabetes mellitus (DM), coronary artery disease, and hypertension (HT). The right trochlear and abducens complete paralysis were present in the patient’s neurological examination (Figures 1a, 1b). Vision loss was not detected, but diplopia was present in the patient upon evaluation by an ophthalmologist. The patient, after consultation with the dermatology department, was diagnosed with HZO. Contrast-enhanced cranial MRI was unremarkable. Lumbar puncture (LP) analysis was within the normal limit. The patient’s well-controlled DM resulted in a hemoglobin A1c (HbA1c) value of 5.6 mmol/mol Hb. The patient was administered methylprednisolone 1mg/kg/day orally and acyclovir 2,400mg/day intravenously for a 14-day period. Follow-ups were performed for six months in regular outpatient clinic control. No improvement was observed in the patient’s extraocular muscle complete paralysis and diplopia during this follow-up.

Case 2

**Figure 2:**
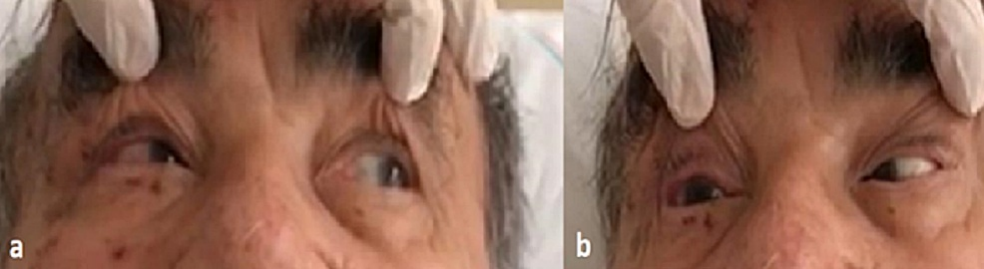
When looking to the right, the eye cannot move outward. Sixth cranial nerve paralysis was observed.

A 76-year-old male patient visited our outpatient clinic because of persistent redness, swelling, and blistering around his right eye for four days. He had a history of HT, DM, and heart failure. Neurological examination showed a limitation of the right eye to move outward (Figures 2a, 2b). Vision loss was not detected, but diplopia was present in the patient upon evaluation by an ophthalmologist. The patient, after consultation with the dermatology department, was diagnosed with ophthalmic shingles. The patient had poor glycemic control and the HbA1c value was 8.4 mmol/mol Hb. Cranial MRI was is unremarkable. The patient, when other possible causes were excluded, was diagnosed with HZO with abducens complete paralysis. Acyclovir was administered intravenously 3000 mg/day for three days, and acyclovir treatment was stopped due to the development of nephropathy secondary to acyclovir treatment. Methylprednisolone was not included in the treatment of the patient with uncontrolled DM. In this patient, when renal functions normalized, brivudine 125 mg/day was administered in consultation with the infectious diseases doctor. Follow-ups were performed for six months in the regular outpatient clinic. Neurological examination showed no improvement was observed in the patient’s extraocular complete paralysis and diplopia during this follow-up.

## Discussion

Herpes zoster usually occurs through the reactivation of varicella-zoster virus in the thoracic and cranial sensory ganglia. When activated in the ophthalmic branch originating from the trigeminal nerve, this condition is called HZO, which occurs in approximately 10% to 25% of herpes zoster cases [1]. HZO occurs at a higher rate than encountered by the normal population, especially in the case of immune-compromised and elderly patients [4]. However, HZO-associated extraocular complete paralysis is uncommon. Isolated trochlear and/or abducens nerve involvement is rare, and therefore it can be easily overlooked during examination [1]. Orbital apex syndrome with combined cranial nerve involvement characterized by dysfunction of the trigeminal nerve ophthalmic branch, oculomotor, trochlear, abducens nerve, and optic nerve has been reported in the literature [5]. Except for orbital apex syndrome, the involvement of the fourth and sixth cranial nerves at the same time is rare. While trochlear and abducens nerves were held together in one of our cases, isolated abducens nerve involvement was observed in the other case. In the literature review, it has been reported that ocular motor paresis of a patient with fourth and sixth cranial nerve involvement improved in approximately two months with treatment [4,6]. Unlike this report, no improvement was observed in extraocular paralysis despite treatment in our cases. When HZO develops, our aims must involve reducing the severity of acute and chronic pain, facilitating rapid recovery, and preventing viral replication [1]. Systemic antivirals and systemic steroids do have a role in the treatment and also prevention of these complications if administered timely, that is, within 72 hours of the rash, which prevents the long-term complications by almost 50%. Patients with HZO are treated with acyclovir orally. The course of treatment ranges from seven to 10 days [4]. Antiviral therapy has been proven to be significantly more effective when it is started within the first 72 hours after the appearance of the rash. Antiviral treatment facilitates rapid regression of skin lesions, reduces viral replication and lesion formation, and prevents cornea and uvea involvement [7]. In addition, some authors have recommended the use of systemic corticosteroids in the treatment plan. It has also been suggested that it may be effective in treating the possible effects of vasculitis and preventing postherpetic neuralgia [5-7]. However, it is also well known that steroids should not be administered in isolation, owing to the risk of triggering viral replication [7]. Although systemic corticosteroid therapy can be effective in preventing obstructive vasculitis, the risk of suppressing the immune system also needs to be considered [1]. In addition to these treatments, the recurrent paravertebral block has been proven to be effective in the treatment of zoster-associated pain [8]. Extraocular muscle palsy associated with HZO is generally known as a transient and self-limiting condition. It has been reported that extraocular muscle complete paralysis associated with HZO develops between one and two weeks after the appearance of skin lesions and usually shows significant improvement within two months. Recovery of diplopia can occur at intervals between two and 23 months [2]. One of our patients was treated with acyclovir and corticosteroids, the other patient was administered only acyclovir, and during six months of follow-ups, no improvement was observed in our two cases. Various pathophysiological theories have been proposed for extraocular muscle paralysis in the literature review [7-10]. De Groot-Mijnes et al. [7] examined 21 enucleated eyes with HZO involvement, and they observed chronic inflammatory cell infiltration. This, in turn, suggested that neuropathy was caused by obstructive vasculitis. While complete recovery was observed in some reported cases, partial recovery was observed in some cases. These differences in prognosis may be attributed to different mechanisms [9,10]. In our cases, we thought that the disruption of nerve nutrition secondary to microangiopathic atherothrombotic vascular pathologies was caused by the patients’ advanced age, and their accompanying comorbidities had a negative effect on the recovery process. Although it generally has a good prognosis according to the literature, the prognosis of extraocular muscle paralysis may be poor in elderly patients with comorbidities.

## Conclusions

Extraocular movements should be carefully examined in patients with HZO. Other possible causes of extraocular muscle palsy should be excluded in etiology, and treatment should be started immediately. Although we paid attention to these, we presented two cases of HZO with poor prognosis and involvement of fourth and sixth cranial nerves palsy. In the presence of extraocular muscle paralysis associated with HZO, which is generally known to have a good prognosis, it should be considered that eye movement may not improve despite adequate treatment in the case of patients with advanced age and multiple comorbid diseases.
